# Metamorphoses

**Published:** 1868-08

**Authors:** 


### METAMORPHOSES.

A LEARNED doctor once delivered himself thus: "Alcohol arrests the metamorphosis of the tissues; hence, as Phthisis is the oxydation of the exudation corpuscle, the article designated should be 'exhibited' in the premises."

Man alive! why didn't you say: "I believe brandy is a cure for consumption."

This grandiloquence is a reminder of another deliverance effected safely by a tip-top preacher, on one of the "squares:" "Anthropology is Theopneustic:" that is, the Wottapottimy man believes in the power of gunpowder. If Drs. Hall,

Davidson, McGill, or any other of our learned D.D.s, will give us a more literal and luminous "rendering," we shall be happy to avail ourselves of their exegetical acumen and learned lore.

No doubt some canny Sawney invented the argument in the *Westminster Review* three years ago, that as alcohol arrested the metemorphosis of the tissues, prevented the waste of flesh and strength of man, hence "Punch" was good for every body, sick or well. The meaning of this is, that we are all the time burning up, the flame must have something to feed upon; that flesh is carbon, whisky is carbon: that carbon only will feed fire, is food for the fire of life, and that, under the circumstances, it is better to furnish whisky, than to supply the demand with ones own flesh. But if you put some "Old Bourbon" in a dish, and set it on fire, it goes off in a blaze, and soon there is but little left beyond a spoonful of water, and if the flame is to be kept up, more whisky must be supplied: hence, who ever heard of a toper who wasn't a personified horse-leech and Oliver Twist "give-more!"

For a time liquor does arrest destructive decay; the fever of disease feeds upon it, instead of the flesh; but then it creates an "excitement," a fever of its own; this, too, must be supplied with fuel: hence, in any given case, either in health or disease, the alcohol must be supplied in increasing quantities, in order to keep the system up to a certain point, and if that increasing quantity is not supplied, the body begins to burn itself up; the man feels wretched; whole pitchers of water are ravenously drank in the vain effort to satiate the remorseless thirst, but an ocean of cold water can't put out the fire; there is a burning fire within the man; it must have flesh to feed it; there is no appetite for the solid food, which otherwise might have formed flesh; the stomach and intestines have been so scorched, as it were, by the immense amount of "fire-water" introduced into them, that they will receive it no longer, or if tolerated, it does not meet the wants of the system, and there is no other alternative but to make fuel of the remnant of flesh yet remaining; pound by pound does that flesh melt away, and the full-fleshed, red-cheeked, hilarious man of a few months ago, has lost his jesting and his jokery, the ruby has faded from his face; on cheek, and arm, and hand, and "calf," the dry and parched skin clings as tightly as a drum-head; he totters along on two spindles; eats nothing, drinks nothing, and miserably dies!

Therefore if any reader of this JOURNAL tries hereafter to get well of any ailment under the sun by drinking liquor, he is so hopelessly stupid, that any additional reading of it will be utterly useless, and he had better write for the balance of his year's subscription.

